# Intranasally Administered Human MSC-Derived Extracellular Vesicles Pervasively Incorporate into Neurons and Microglia in both Intact and Status Epilepticus Injured Forebrain

**DOI:** 10.3390/ijms21010181

**Published:** 2019-12-26

**Authors:** Maheedhar Kodali, Olagide W. Castro, Dong-Ki Kim, Alicia Thomas, Bing Shuai, Sahithi Attaluri, Raghavendra Upadhya, Daniel Gitai, Leelavathi N. Madhu, Darwin J. Prockop, Ashok K. Shetty

**Affiliations:** 1 Institute for Regenerative Medicine, Department of Molecular and Cellular Medicine, Texas A&M University College of Medicine, 1114 TAMU, College Station, TX 77842, USA; kodali@tamu.edu (M.K.); biodon2003@msn.com (D.-K.K.); aliciathomas65@gmail.com (A.T.); shuai@medicine.tamhsc.edu (B.S.); sahithi.attaluri@tamu.edu (S.A.); sacraghu@tamu.edu (R.U.); lnmadhu802@tamu.edu (L.N.M.); prockop@tamu.edu (D.J.P.); 2 Health Science Center, Federal University of Alagoas, Maceio 57072900, Brazil; olagidewww@gmail.com (O.W.C.); danielgitai@gmail.com (D.G.)

**Keywords:** brain injury, exosomes, extracellular vesicles, intranasal administration, mesenchymal stem cells, microglia, microvesicles, status epilepticus, transport of extracellular vesicles

## Abstract

Extracellular vesicles (EVs) derived from human bone marrow mesenchymal stem cells (hMSCs) have great promise as biologics to treat neurological and neurodegenerative conditions due to their robust antiinflammatory and neuroprotective properties. Besides, intranasal (IN) administration of EVs has caught much attention because the procedure is noninvasive, amenable for repetitive dispensation, and leads to a quick penetration of EVs into multiple regions of the forebrain. Nonetheless, it is unknown whether brain injury-induced signals are essential for the entry of IN-administered EVs into different brain regions. Therefore, in this study, we investigated the distribution of IN-administered hMSC-derived EVs into neurons and microglia in the intact and status epilepticus (SE) injured rat forebrain. Ten billion EVs labeled with PKH26 were dispensed unilaterally into the left nostril of naïve rats, and rats that experienced two hours of kainate-induced SE. Six hours later, PKH26 + EVs were quantified from multiple forebrain regions using serial brain sections processed for different neural cell markers and confocal microscopy. Remarkably, EVs were seen bilaterally in virtually all regions of intact and SE-injured forebrain. The percentage of neurons incorporating EVs were comparable for most forebrain regions. However, in animals that underwent SE, a higher percentage of neurons incorporated EVs in the hippocampal CA1 subfield and the entorhinal cortex, the regions that typically display neurodegeneration after SE. In contrast, the incorporation of EVs by microglia was highly comparable in every region of the forebrain measured. Thus, unilateral IN administration of EVs is efficient for delivering EVs bilaterally into neurons and microglia in multiple regions in the intact or injured forebrain. Furthermore, incorporation of EVs by neurons is higher in areas of brain injury, implying that injury-related signals likely play a role in targeting of EVs into neurons, which may be beneficial for EV therapy in various neurodegenerative conditions including traumatic brain injury, stroke, multiple sclerosis, and Alzheimer’s disease.

## 1. Introduction

Extracellular vesicles (EVs) are nanosized membranous particles released by cells into the extracellular space [1]. EVs carry a cargo of nucleic acids and proteins and are delimited by a phospholipid bilayer [2]. Microvesicles (MVs, 100–1000 nm) that bud off directly from the plasma membrane, and exosomes (EXs, 30–150 nm) originating in endosomes and then secreted from multivesicular bodies into the extracellular space, are the two major types of EVs [3,4]. Because of some overlap in size as well as markers between MVs and EXs, the phrase “EVs” is now widely employed for all vesicles secreted by cells, unless the MV or EX identity is available through live imaging techniques [1]. Intercellular communication is the primary function of EVs through which they likely influence a variety of cellular processes [1,2]. Recent reports have shown that EVs mediate cell-cell communication by transferring proteins, mRNAs, and microRNAs to target cells [5,6]. Therefore, characterization of proteins and/or microRNAs in EVs in the serum or the cerebrospinal fluid (CSF) have provided information on specific biomarkers in various neurodegenerative diseases [7,8]. Notably, early markers of CNS diseases can be gleaned from the characterization of neuron-, astrocyte-, oligodendrocyte- or microglia-derived EVs in the serum or the CSF. The examples of biomarkers identified from EVs in the serum or CSF include higher levels of specific miRNAs in amyotrophic lateral sclerosis [9] and traumatic brain injury [10], altered lysosomal and synaptic proteins and tau in Alzheimer’s disease (AD) [11,12,13,14], increased concentration of alpha-synuclein in Parkinson’s disease [15], and HMGB1 and complement-related proteins in an animal model of Gulf War Illness [16].

On the other hand, EVs secreted by mesenchymal stem cells or neural stem cells have therapeutic properties [17,18]. A series of studies have uncovered the curative effects of human mesenchymal stem cell (hMSC)-derived EVs, for treating a variety of neurological conditions. The beneficial effects of MSC-derived EVs were observed in animal models of traumatic brain injury (TBI) [19,20], stroke or ischemia [21,22,23], status epilepticus-induced brain injury [24], multiple sclerosis [25], Alzheimer’s disease (AD) [26], and spinal cord injury [27]. For example, intravenous (IV) administration of hMSC-EVs an hour after the induction of controlled cortical impact injury was efficient for suppressing the early surge of proinflammatory cytokines as well as easing TBI-induced cognitive impairments [20]. Likewise, intranasal (IN) administration of hMSC-EVs after two hours of status epilepticus (SE) was efficacious for significantly reducing SE-induced glutamatergic excitatory and GABA-ergic inhibitory neuron loss, acute and chronic neuroinflammation as well as abnormal neurogenesis [24].

Thus, therapeutic benefits have been seen with EV administration through both IV and IN routes in animal models of brain injury. However, it is believed that the IN route is more efficient for directing a large proportion of administered EVs quickly into the brain, which may help in reducing the dose required for inducing functional recovery in neurological disease conditions. Besides, the IN route is non-invasive, allows repetitive administration, and amenable for developing the administration of EVs through a nasal spray in the future. Nonetheless, it is unknown whether IN-administered EVs pervasively penetrate all regions of the entire forebrain or they require brain injury-induced signals for entry into specific brain regions. Therefore, in this study, we examined the targeting of IN-administered hMSC-derived EVs into neurons and microglia in different regions of the intact or status epilepticus (SE) injured rat forebrain. Our results provide novel evidence that unilateral IN administration of EVs is adequate for dispensing EVs bilaterally into neurons and microglia in virtually every region of the intact or injured forebrain. Our results also showed that the incorporation of EVs by neurons is higher in areas of brain injury, implying the role of injury-related signals in targeting of EVs into neurons.

## 2. Results

### 2.1. IN-Administered PKH26-Labeled hMSC-EVs Pervasively Penetrated all Regions of the Intact and SE-Injured Forebrain

**Figure 1:**
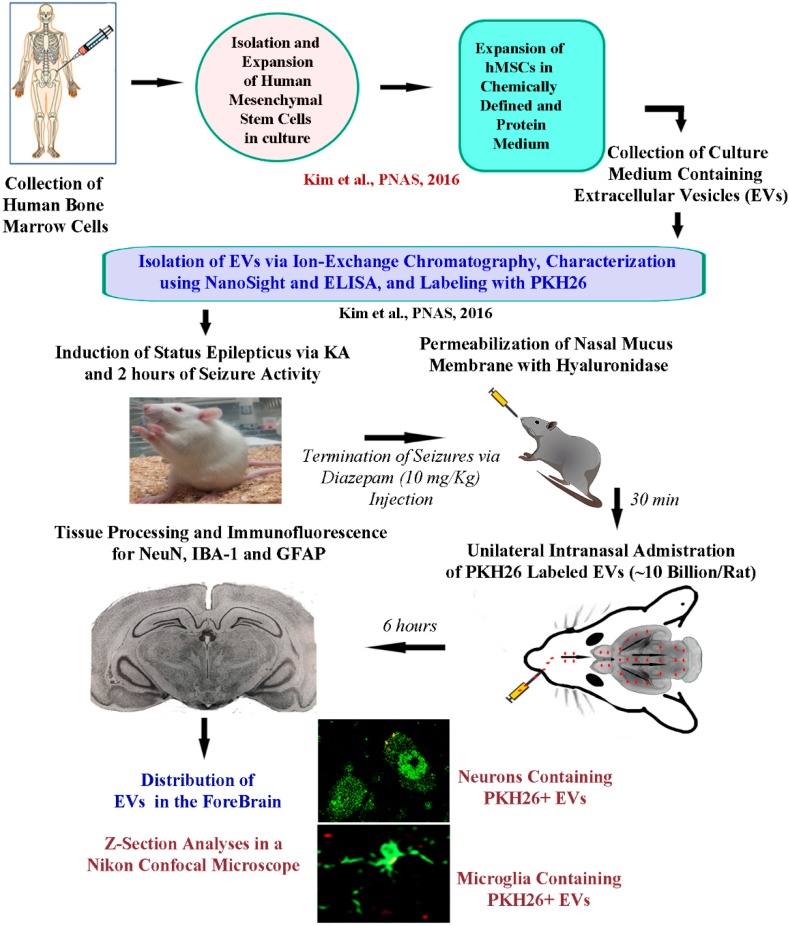
A schematic to show the timeline of experiments and analyses. The human bone marrow-derived mesenchymal stem cells (hMSCs) were expanded in a chemically defined and protein medium. The culture supernatant comprising extracellular vesicles (EVs) derived from hMSCs was collected. The EVs were next isolated by ion-exchange chromatography and labeled with PKH26. The EVs were characterized for EV markers such as CD63 using ELISA, and the size of EVs was evaluated by nano tracking analysis using NanoSight. To study the proficiency of EVs to target neural cells in various regions of the intact and injured forebrain, intranasal (IN) administration of PKH26-labeled EVs was performed to five-week-old naïve rats and rats that underwent 2 h of status epilepticus (SE). SE was induced through graded injections of kainic acid, and seizures were terminated by subcutaneous injection of diazepam (10 mg/kg). Thirty minutes before the administration of EVs (10 billion/rat), the permeability of the nasal mucous membrane was enhanced by hyaluronidase treatment. IN administration of EVs was performed unilaterally through the left nostril. Six hours later, the rats were perfused, and brain tissue sections were processed for immunofluorescent studies to analyze the biodistribution of EVs in neurons, microglia, and astrocytes, in multiple regions of the forebrain.

The research design employed in the study is illustrated in Figure 1. Briefly, the isolation and expansion of human bone marrow-derived MSCs in culture, isolation, and characterization of EVs were done as described in our previous reports [20,24]. Induction of SE in rats was performed using procedures detailed in previous reports [28,29,30]. IN administration of 10 billion EVs was performed 2.5 h after the induction of SE following permeabilization of the nasal mucous membrane with hyaluronidase for 30 min (Figure 1). Animals were euthanized through intracardiac perfusion with paraformaldehyde at 6 h after the administration of EVs and brain tissues were processed for sectioning at thirty-micrometer thickness using a cryostat. Next, the EVs were examined in a confocal microscope following immunofluorescence staining for neuron-specific nuclear antigen (NeuN), ionized calcium-binding adapter molecule 1 (IBA-1), or glial fibrillary acidic protein (GFAP) (Figure 1).

**Figure 2:**
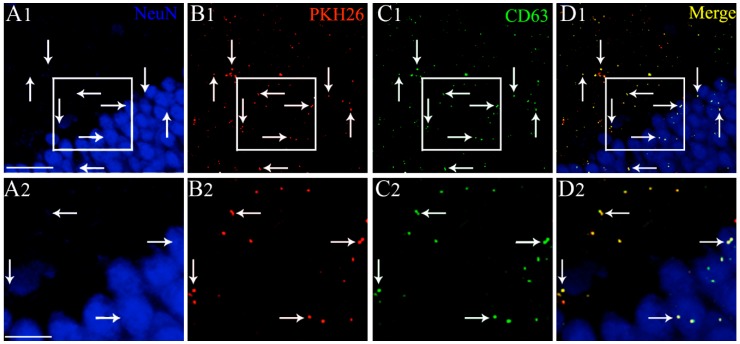
Intranasally administered PKH26-labeled extracellular vesicles (EVs) found in the forebrain expressed the EV marker CD63, at 6 h post-administration. An example from the dentate gyrus (DG) of the hippocampus is illustrated (**A1**, DG). Please note that intranasally administered PKH26-labeled extracellular vesicles (EVs) (**B1**, red dots) found within the soma or in close contact with the cell membrane of NeuN + neurons (blue) express CD63 (**C1**, green dots). **D1** illustrates the merged image exhibiting the expression of both PKH26 and CD63 in EVs (yellow dots). (**A2**,**B2**,**C2**,**D2**) are magnified views of boxed regions in (**A1**,**B1**,**C1**,**D1**). Scale bars: (**A1**,**B1**,**C1**,**D1**) 50 µm; (**A2**,**B2**,**C2**,**D2**) 25 µm.

Examination of serial sections through the forebrain revealed the presence of PKH26 labeled EVs in virtually all regions of the forebrain in both naïve animals and animals that underwent 2 h of SE. We found a similar distribution of EVs in both hemispheres. Therefore, we randomly picked various forebrain regions from one of the hemispheres and quantified the distribution of EVs in neurons and microglia. In both groups, EVs were found inside neuron-specific nuclear antigen (NeuN) positive neurons and IBA-1 + microglia. EVs were also found in contact with the processes of GFAP + astrocytes, as described in our previous study [24]. To authenticate that the PKH26 + particles found inside neurons are EVs, we processed brain tissue sections for NeuN and CD63 dual immunofluorescence staining and examined the co-expression of CD63 with PKH26 + particles. Our analysis revealed that all PKH26 + particles, including those found inside NeuN + neurons, were also positive for CD63. Examples of IN-administered EVs targeting the dentate gyrus of the hippocampus and expressing both PKH26 and CD63 are illustrated in Figure 2. The tetraspanin CD63, a member of the transmembrane 4 superfamily and typified by the manifestation of four hydrophobic domains, is a specific marker of EVs. Because CD63 is expressed in both endosome-derived exosomes and plasma membrane-derived microvesicles, our results confirmed that PKH26 + particles found inside neural cells are indeed EVs. Since EVs were mostly found inside neurons and microglia but not within astrocytes, additional quantitative studies on EV incorporation were focused on neurons and microglia.

### 2.2. A Higher Percentage of Neurons in the CA1 Subfield of the Hippocampus Internalized hMSC-EVs in Animals that Underwent SE

**Figure 3:**
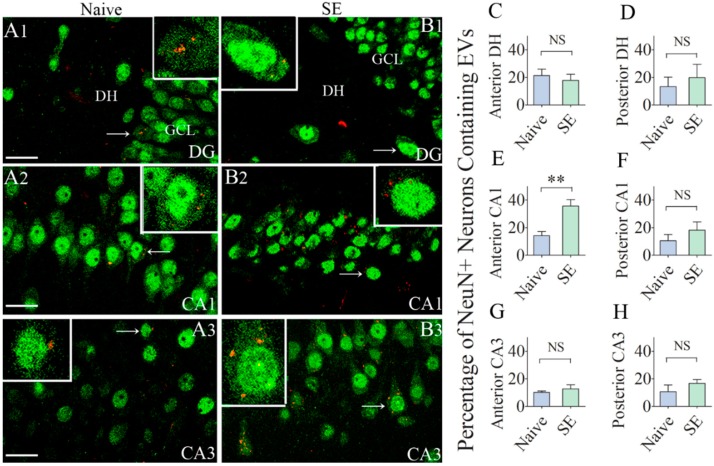
Significant percentages of NeuN + neurons in the anterior and posterior regions of the hippocampus internalize the intranasally administered PKH26-labeled EVs by 6 h. Figures (**A1**–**B3**) illustrate the presence of PKH26-labeled EVs (red/yellow dots) within the cytoplasm or in close contact with the cell membrane of NeuN + neurons in the dentate hilus (DH, **A1**,**B1**), the **CA1** pyramidal cell layer (**A2**,**B2**) and the **CA3** pyramidal cell layer (**A3**,**B3**), of the hippocampus from a naïve control rat (**A1**–**A3**) and a rat that underwent status epilepticus (SE; **B1**–**B3**). Insets show magnified views of individual neurons containing PKH26 + EVs. The bar charts in (**C**–**H**) compare percentages of EVs targeting neurons in different regions of the hippocampus between naïve rats and rats that underwent SE. NS, not significant, ** *p* < 0.01, Scale bars: A1–B3, 20 µm.

Since the hippocampus is the susceptible region for neurodegeneration after SE, we first examined the incorporation of hMSC-EVs in neurons belonging to different subfields of the hippocampus. We found EVs inside a significant percentage of neurons in the dentate hilus, and CA1 and CA3 pyramidal cell layers in both naïve animals and animals that underwent SE (Figure 3(A1–B3)). Most EVs were seen in smaller clusters within the cytoplasm or attached to the cell membrane of neurons in both the intact and injured hippocampus (Figure 3(A1–B3)). Quantification of the percentages of neurons incorporating EVs revealed that comparable percentages of neurons internalized EVs in the dentate hilus, the posterior half of the CA1 subfield, and the CA3 subfield (10–21%, *p* > 0.05, Figure 3C,D,F–H. However, in the anterior half of the CA1 subfield, a significantly higher percentage of CA1 pyramidal neurons (36%) incorporated EVs in animals that underwent SE, in comparison to only 14% of CA1 pyramidal neurons incorporating EVs in naïve control animals (*p* < 0.01, Figure 3E). Thus, after SE, only the CA1 pyramidal neurons in the hippocampus displayed a higher percentage of EV intake.

### 2.3. IN-Administered EV Incorporation by Neurons Were Comparable between Naïve and SE Rats in most Regions of the Cerebral Cortex, Thalamus and Striatum

**Figure 4:**
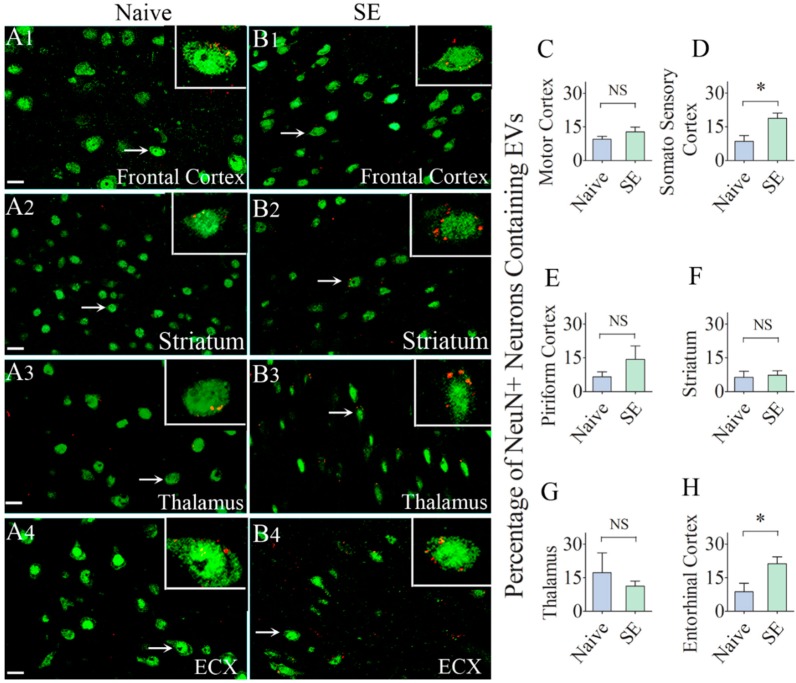
Significant percentages of NeuN + neurons in different regions of the forebrain incorporate the intranasally administered PKH26-labeled EVs by 6 h. The figures (**A1**–**B4**) illustrate the presence of PKH26-labeled EVs (red/yellow dots) within the cytoplasm or in close contact with the cell membrane of NeuN + neurons in the Frontal Cortex (**A1**,**B1**), Striatum (**A2**,**B2**), Thalamus (**A3**,**B3**) and Entorhinal Cortex (ECX,**A4**,**B4**) of naïve control rats (**A1**–**A4**) and rats that underwent status epilepticus (SE, B1–B4). Insets show magnified views of individual neurons containing PKH26 + EVs. The bar charts in (**C**–**H**) compare percentages of EVs targeting neurons in different regions of the forebrain between naïve rats and rats that underwent SE. NS, not significant, *, *p* < 0.05, Scale bars: (**A1**–**B4**), 20 µm.

Next, we probed whether IN administration of hMSC-EVs would also result in their entry into neurons in extrahippocampal regions of the forebrain. We found the internalization of EVs by a significant percentage of neurons in virtually every region of the forebrain in both naïve rats and rats that underwent SE. The examined regions of the cerebral cortex included the olfactory cortex, motor cortex, somatosensory cortex, piriform cortex, insular cortex, subiculum, and entorhinal cortex. The deep forebrain regions examined comprised the striatum, septum, thalamus, hypothalamus, and amygdala. Representative images displaying EV incorporation by neurons in the frontal cortex, striatum, thalamus, and the entorhinal cortex of naïve and SE rats are illustrated in Figure 4. Quantification of the percentage of neurons incorporating EVs in the motor cortex, piriform cortex, striatum, and thalamus revealed no differences between naïve rats and rats that underwent SE (Figure 4C,E–G). However, a higher percentage of neurons in the somatosensory cortex (19% and entorhinal cortex (21%) incorporated EVs in rats that underwent SE, in comparison to their counterparts in naïve control animals (9%, *p* < 0.05, Figure 4D,H). Cumulatively, by 6 h after IN administration, EVs incorporated robustly into neurons in virtually all regions of the cerebral cortex as well as the deep forebrain areas. SE-induced brain injury increased the intake of EVs by neurons in the somatosensory cortex and the entorhinal cortex.

### 2.4. Comparable Percentages of Microglia Incorporated IN-Administered EVs in different Subfields of the Hippocampus

**Figure 5:**
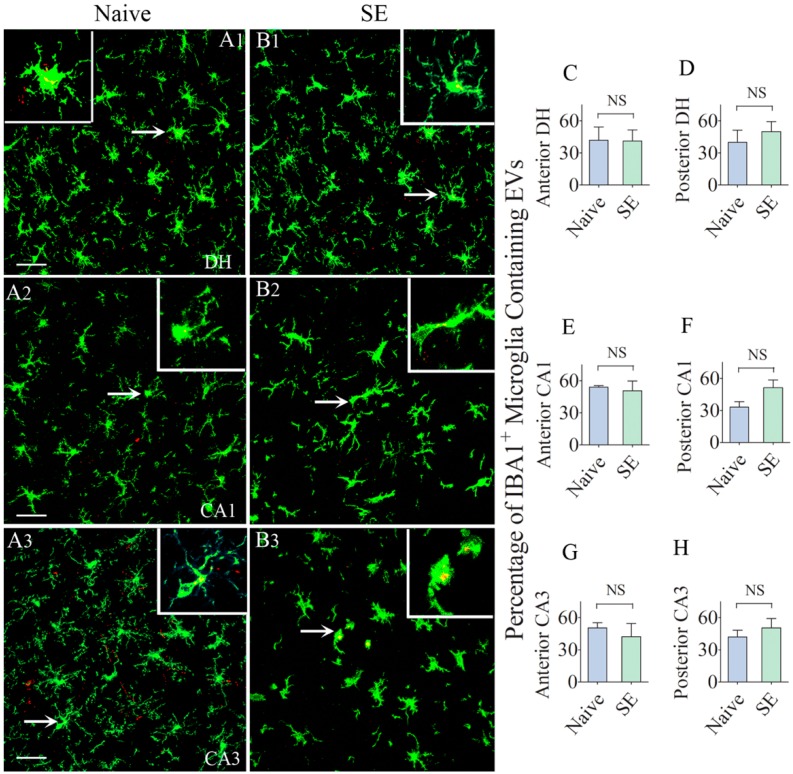
Higher percentages of IBA-1 + microglia in the anterior and posterior regions of the hippocampus take up the intranasally administered PKH26-labeled EVs by 6 h. The figures **A1**–**B3** show the occurrence of PKH26-labeled EVs (yellow-colored structures) within the soma or processes of IBA-1 + microglia in the dentate hilus (DH, **A1**,**B1**), and the CA1 and CA3 subfields (**A2**,**B2**,**A3**,**B3**) of the hippocampus in a naïve control rat (**A1**–**A3**) and a rat that underwent status epilepticus (SE,B1-B3). Insets show magnified views of individual microglia containing clusters of PKH26 + EVs. The bar charts in **C**–**H** compare percentages of EVs targeting IBA-1 + microglia in different regions of the hippocampus between naïve rats and rats that underwent SE. NS, not significant, Scale bars: (**A1**–**B3**), 20 µm.

We first examined the intake of EVs by IBA-1 + microglia in the intact and SE-injured hippocampus. We found EVs inside significant percentages of microglia in the dentate hilus, and CA1 and CA3 subfields in both groups (Figure 5(A1–B3)). Larger clusters of EVs were seen in the cytoplasm of the cell body of microglia whereas, smaller clusters of EVs were conspicuous in the processes of the microglia (Figure 5(A1–B3)). Qualitatively, the size of EV clusters appeared similar between naïve rats and rats that underwent SE. Quantification also revealed no differences in the percentage of microglia incorporating EVs between naïve rats and rats that underwent SE in all subfields (40–54%, Figure 5C–H. Thus, the intake of EVs by microglia was highly comparable between the naïve hippocampus and the SE-injured hippocampus. However, in comparison to the incorporation of EVs by neurons in most regions of the hippocampus (10–21%), more significant percentages of microglia (40–54%) in both naïve and the injured hippocampus incorporated EVs.

### 2.5. Similar Percentages of Microglia Internalized IN-Administered EVs in Different Regions of the Cerebral Cortex and Deep Forebrain Areas

**Figure 6:**
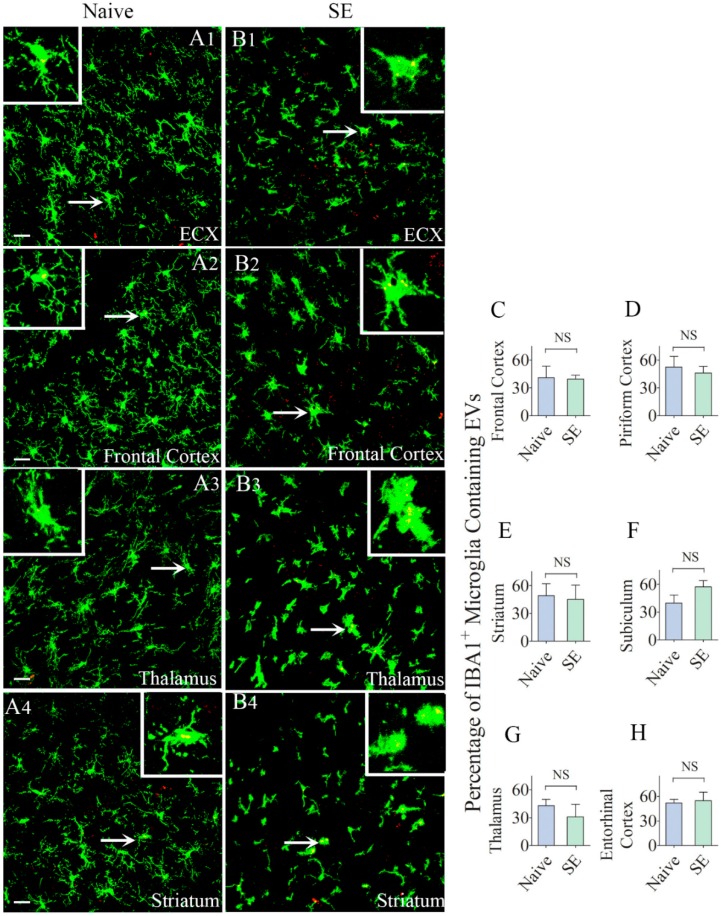
Higher percentages of IBA-1 + microglia in different regions of the forebrain internalize the intranasally administered PKH26-labeled EVs by 6 h. The figures (**A1**–**B4**) show the presence of PKH26-labeled EVs (yellow-colored structures) within the soma or processes of IBA-1 + microglia in the Entorhinal Cortex (ECX;**A1**,**B1**), Frontal Cortex (**A2**,**B2**), Striatum (**A3**,**B3**) and Thalamus (**A4**,**B4**) of naïve control rats (**A1**–**A4**) and rats that underwent status epilepticus (SE,B1–B4)). Insets show magnified views of individual microglia containing clusters of PKH26 + EVs. The bar charts in (**C**–**H**) compare percentages of EVs targeting IBA-1 + microglia in different regions of the hippocampus between naïve rats and rats that underwent SE. NS, not significant, Scale bars: (**A1**–**B3**), 20 µm.

We also examined whether IN administration of hMSC-EVs would also result in their entry into microglia located in multiple regions of the forebrain. We found the internalization of EVs by microglia in every region of the forebrain we examined in both naïve rats and rats that underwent SE. The regions investigated in the cerebral cortex comprised the olfactory cortex, motor cortex, somatosensory cortex, piriform cortex, insular cortex, subiculum, and entorhinal cortex. The deep forebrain regions analyzed encompassed the striatum, septum, thalamus, hypothalamus, and amygdala. Demonstrative pictures displaying EV intake by microglia in the entorhinal cortex, frontal cortex, thalamus, and striatum of naïve and SE rats are illustrated in Figure 6. Measurement of the percentage of microglia internalizing EVs in the frontal cortex, piriform cortex, striatum, subiculum, thalamus, and entorhinal cortex revealed no differences between naïve rats and rats that underwent SE (39–57%, Figure 6C–H). Thus, by 6 h after IN administration, EVs incorporated robustly into microglia in virtually all regions of the cerebral cortex as well as the deep forebrain areas. SE-induced brain injury did not increase the intake of EVs by microglia in any region of the forebrain; however.

## 3. Discussion

The results of this study underscore that unilateral IN administration of hMSC-EVs is adequate for delivering EVs bilaterally into neurons and microglia in multiple regions of the intact or injured forebrain. Analyses of the percentage of neurons incorporating EVs in different regions of the SE-injured brain further revealed that a more significant percentage of neurons tend to internalize EVs in brain areas that are susceptible to neurodegeneration following insults such as SE. The suitability of the IN route for delivering EVs generated from different cell types into the brain has also been observed in several previous studies. Specifically, IN administration of different types of EVs or EVs loaded with beneficial proteins reduced neuroinflammation in a model of experimental autoimmune encephalomyelitis [31], mediated neuroprotection in models of Parkinson’s disease [32] and ischemic-reperfusion injury [33], and reduced neurodegeneration and neuroinflammation as well as prevented cognitive dysfunction in a model status epilepticus [24]. The results of the current study demonstrated that IN administration of EVs is the most efficient approach for delivering EVs into virtually all regions of the forebrain. These observations have considerable importance because earlier studies have shown that systemically administered EVs accumulate mostly in the liver and the spleen [34,35,36].

Interestingly, in the current study, percentages of neurons incorporating IN-administered EVs were comparable between naïve control animals and animals that underwent SE-induced brain injury in most regions of the forebrain, when examined 6 h after IN administration. This finding supports the notion that IN-administered EVs quickly enter different regions of the intact and SE-injured forebrain through similar mechanisms. It is plausible that IN administration of EVs, as performed in the current study, allowed the entry of EVs into the subarachnoid space along olfactory nerves passing through the cribriform plate. Indeed, a communication exists between the nasal lymph compartment and the brain’s CSF compartment [37]. It was suggested that CSF passes through the cribriform plate into lymphatic vessels located in the submucosa associated with the olfactory and respiratory epithelium. Also, the lymph within the ethmoidal lymphatics is believed to be continuous with CSF in the perineurial spaces associated with the olfactory nerves. Additional observations suggest the presence of multiple lymphatic ducts forming a collar around the emerging nerve roots, which facilitate a direct connection between CSF and lymph in the olfactory submucosa [38]. Once EVs enter the CSF, they can be transported via CSF flow in the subarachnoid space, which likely facilitates their entry into the entire surface of the brain as well as brain ventricles. However, it remains to be investigated how EVs penetrate deep into the brain. Since EVs are capable of passing through cells by a process called transcytosis [39], it is not surprising that they penetrated deeper regions of the forebrain by 6 h. It was suggested that transcellular transport of EVs through endothelial cells is mediated by the endothelial recycling endocytic pathway [39].

Regardless of the underlying mechanisms, the current study demonstrated that the IN route is also efficient for delivering EVs into virtually all regions of the intact adult forebrain. This observation supports the possibility of employing IN administration of EVs as a prophylactic neuroprotective treatment for conditions such as aging to combat the low-level chronic neuroinflammation causing mild cognitive impairment. Such an approach may serve as a preventive measure against the development of AD in old age. On the other hand, higher percentages of neurons incorporating EVs in brain areas undergoing neurodegeneration is likely also beneficial for reducing neurodegeneration. In this study, higher percentages of neurons incorporated EVs in the CA1 subfield, and the entorhinal cortex of animals that underwent SE. Both of these regions are known to display neurodegeneration after SE [28,29,40,41,42,43,44]. Therefore, it may be that injury-related signals play a role in targeting of EVs into neurons in the injured brain. This feature would be beneficial for the application of EV therapy in various neurodegenerative conditions, including TBI, stroke, multiple sclerosis, and AD. However, it cannot be ruled out that EV incorporation by neurons would vary depending on the timing of their administration. In this study, EVs were administered early (~2 h) after the induction of SE, and hence injury-induced signals might be low at this timepoint. Additional studies employing IN administration of EVs at multiple timepoints after SE will be needed to fully understand the intake of EVs by neurons in different regions of the injured forebrain.

Another observation in this study is that higher percentages of microglia incorporated EVs than neurons in both intact and the injured brain. Also, relatively larger clusters of EVs accumulated within microglia, in comparison to EV incorporation in neurons. Such discrepancy might be because incorporation of EVs by microglia occurs through macropinocytosis [45] or phagocytosis [46] whereas, the uptake of EVs by neurons is mediated through clathrin-mediated endocytosis [47]. Macropinocytosis or “cell drinking” or bulk endocytosis is associated with the actin-dependent formation of membrane ruffles (lamellipodia), resulting in the uptake of fluid into large vacuoles. Membrane ruffling and closure are dependent on Rac1 GTPase activity, Na^+^/H^+^ exchanger function, and in some cells, on dynamin [45,48,49]. Whereas, phagocytosis is particle driven and depends on the tight interaction of ligands on the particle with receptors on the entire surface of the enclosing cup. A higher level of incorporation of EVs by microglia may be due to the possibility that macropinocytosis and phagocytosis can occur faster and internalize more EVs than clathrin-mediated endocytosis, which involves relatively slow kinetics [50,51].

In summary, the results of this study provide the novel evidence that IN dispensation of EVs is an efficient approach for targeting EVs into significant percentages of neurons and microglia in all regions of both intact and injured forebrain. Higher dose administration of EVs may further increase the percentage of neurons and microglia incorporating EVs. However, it remains to be investigated whether EVs would display a similar pattern of targeting into neurons and microglia in conditions presenting widespread neurodegeneration and neuroinflammation, such as in the later stages of the AD.

## 4. Materials and Methods

### 4.1. Animals

We employed young adult (5 weeks old) male F344 rats acquired from Harlan (Harlan, Indianapolis, IN, USA) in this study. Animals were housed in an environmentally controlled room with a 12:12-h light: dark cycle and were given food and water ad *libitum.* Approximately 7–10 days after their arrival to the vivarium, the animals were randomly assigned to naïve control or SE groups. All experimental procedures were conducted as per the guidelines set by the Animal Care and Use Committee of the Texas A&M University, which is compliant with all applicable federal and state regulations for the purchase, transportation, housing, and ethical use and euthanasia of the animals used for research.

### 4.2. Study Design

The timeline of various experiments is illustrated in Figure 1. The initial experimental steps comprised the preparation of hMSC-EVs, the induction and maintenance of SE for 2 h, permeabilization of the nasal mucous membrane through hyaluronidase treatment to the left nostril (i.e., 2 h after SE induction). Additional steps comprised intranasal administration of EVs (30 min after hyaluronidase treatment), and the timepoint of analysis of the distribution of EVs in different regions of the forebrain (6 h after the IN administration).

### 4.3. hMSC Cultures, Isolation and Labeling of EVs

The protocols for preparation, selection, characterization, culture, isolation, and labeling of EVs from hMSCs are detailed in our previous reports [20,24]. Briefly, the complete culture medium (CCM) from the passage-4 hMSC cultures was replaced with a fresh CCM after 2–3 days. Once the cells attained ~70% confluence (between 4 and 6 days), the CCM was replaced with a commercial medium (CD-CHO medium; cat. no. 10743-002; Invitrogen) optimized with supplements. The spent medium harvested at 6–48 h of culturing hMSCs was employed for EVs isolation. The harvested spent medium was pooled and stored at -80 degrees. After passive thawing, the medium was centrifuged at 2565× *g* for 15 min to remove cellular debris. The supernatant collected was subjected to isolation of EVs by ion-exchange chromatography, as described in our previous reports [20,24]. The isolated EVs were first evaluated for their anti-inflammatory activity in a model of lipopolysaccharide (LPS) induced inflammation as described elsewhere [20]. EVs that showed significant reduction (*p* < 0.05) in the concentration of proinflammatory markers (IL-6, IFN- γ, and IL-1 β) in spleen tissues after intravenous injection of LPS were included for studies. Batches of EVs that did not decrease the levels of proinflammatory cytokines were excluded. To track the EVs in different regions of the brain, the isolated EVs were labeled with the red fluorescent membrane dye PKH26 (MINI26; Sigma, St. Louis, MO, USA), centrifuged and filtered as described elsewhere [20,24]. The lipophilic dye, PKH 26 is a fluorochrome in the red spectrum with peak excitation (551 nm) and emission (567 nm) properties corresponding with rhodamine recognition. Incubation of EVs with PKH26 leads to the incorporation of aliphatic reporter molecules into the lipid bilayer of the extracellular vesicle.

### 4.4. Induction of SE in F344 Rats 

After a week of acclimatization in the vivarium, SE was induced in the rats via graded hourly intraperitoneal injections of kainic acid (KA, 5 mg/Kg) for 2–5 h until they displayed either a state of continuous stage 4 seizures characterized by bilateral forelimb clonus with signs of rearing, or a first stage 5 seizure typified by bilateral forelimb clonus with rearing and falling followed by continuous stages 3–5 seizures for over 10 min [28,29,30,42,44]. Rats continuously displayed stages of III–V seizures for two hours after the onset of SE. The behavioral seizures were terminated at 2 h after the first stage IV/V seizure through subcutaneous diazepam (10 mg/Kg) injection. Animals that did not meet the criteria (either died or did not develop SE) were excluded from the study.

### 4.5. Intranasal Administration of EVs 

Both naïve rats (*n* = 6) and rats underwent SE (*n* = 6) received IN administration of hMSC-EVs. In the SE group, only those animals that displayed SE for 2 h were employed. In each animal, the left nostril was treated with 10 μL of hyaluronidase (100 U; H3506; Sigma-Aldrich) in sterile PBS solution to enhance the permeability of the nasal mucous membrane. Thirty minutes later, each rat was gently held with the ventral side up, and the head was facing downward for the intranasal (IN) administration of EVs or PBS. EVs were suspended in sterile PBS solution at a concentration of 200 μg/mL and stored at −80 °C. Approximately 10 billion EVs labeled with PKH26 were dispensed unilaterally into the left nostril of each rat in 5-μL spurts separated by 5 min.

### 4.6. Tissue Processing and Immunofluorescence Studies

Six hours after SE, animals in both naïve and SE groups underwent intracardiac perfusions with 4% paraformaldehyde. The brains were dissected, post-fixed in 4% paraformaldehyde overnight, and processed for sectioning using a cryostat [52,53,54,55,56]. Thirty micrometer-thick coronal sections were cut through the entire forebrain and collected serially in 24-well plates containing the phosphate buffer. Several sets of serial sections through the entire brain were selected and processed for immunofluorescent staining using appropriate primary antibodies against NeuN (a neuronal marker; Millipore), IBA-1 (a microglia marker; Abcam) and GFAP (a marker of astrocytes, DAKO) [16,24,52] Matching secondary antibodies conjugated to fluorescent probes purchased from either Jackson ImmunoResearch or Thermo Fisher Scientific were employed. Sections were mounted by using an antifade-slow fade reagent (Sigma, St. Louis, MO, USA).

### 4.7. Confocal Microscopy

Optical Z-sections (1-μm thick) were sampled from different forebrain regions using a Nikon confocal microscope [53,54,55,56]. Z-stacks were captured from three different fields in every region of the forebrain for every marker analyzed in the study. Four naïve control rats and four rats that underwent SE were randomly chosen for quantitative analysis of EV incorporation by neurons and microglia (*n* = 4/group). The regions of the forebrain imaged comprised the frontal cortex, olfactory cortex, motor cortex, piriform cortex, somatosensory cortex, insular cortex and the entorhinal cortex, different subfields (dentate gyrus, and CA1 and CA3 subfields) of the hippocampus, subiculum, striatum, septum, amygdala, thalamus and the hypothalamus. The images were used for quantifying the percentage of neurons or microglia that incorporated EVs using the NIS elements image browser.

### 4.8. Statistical Analyses

The results were compared statistically using the Prism software. Data from naïve rats and rats that underwent SE were compared using an unpaired, two-tailed, Student’s *t*-test. The differences were considered statistically significant when *p* < 0.05.
